# Modelling the pathogenesis of X-linked distal hereditary motor neuropathy using patient-derived iPSCs

**DOI:** 10.1242/dmm.041541

**Published:** 2020-01-13

**Authors:** Gonzalo Perez-Siles, Anthony Cutrupi, Melina Ellis, Jakob Kuriakose, Sharon La Fontaine, Di Mao, Motonari Uesugi, Reinaldo I. Takata, Carlos E. Speck-Martins, Garth Nicholson, Marina L. Kennerson

**Affiliations:** 1 Northcott Neuroscience Laboratory, ANZAC Research Institute, Sydney, 2139 NSW, Australia; 2 Sydney Medical School, University of Sydney, Sydney, 2050 NSW, Australia; 3 School of Life Sciences, University of Technology Sydney, Sydney, 2007 NSW, Australia; 4 Centre for Cellular and Molecular Biology, School of Life and Environmental Sciences, Deakin University, Burwood, 3125 VIC, Australia; 5 Institute for Integrated Cell-Material Sciences and Institute for Chemical Research, Kyoto University, Kyoto 606-8302, Japan; 6 Sarah Network Rehabilitation Hospitals, Brasilia, 70297-400 DF, Brazil; 7 Molecular Medicine Laboratory, Concord Repatriation General Hospital, Sydney, 2139 NSW, Australia

**Keywords:** *ATP7A*, Copper, Induced pluripotent stem cell, Motor neurons, dHMN

## Abstract

*ATP7A* encodes a copper-transporting P-type ATPase and is one of 23 genes in which mutations produce distal hereditary motor neuropathy (dHMN), a group of diseases characterized by length-dependent axonal degeneration of motor neurons. We have generated induced pluripotent stem cell (iPSC)-derived motor neurons from a patient with the p.T994I *ATP7A* gene mutation as an *in vitro* model for X-linked dHMN (dHMNX). Patient motor neurons show a marked reduction of ATP7A protein levels in the soma when compared to control motor neurons and failed to upregulate expression of ATP7A under copper-loading conditions. These results recapitulate previous findings obtained in dHMNX patient fibroblasts and in primary cells from a rodent model of dHMNX, indicating that patient iPSC-derived motor neurons will be an important resource for studying the role of copper in the pathogenic processes that lead to axonal degeneration in dHMNX.

## Abstract (precis)

**Summary:** The authors describe a neuronal model to investigate how mutations in the copper transporter ATP7A cause axonal degeneration in the peripheral nervous system.

## INTRODUCTION

Mutations in the copper (Cu)-transporting P-type ATPase *ATP7A* gene cause three distinct human diseases: Menkes disease (MD) (Mercer et al., 1993; Chelly et al., 1993; Vulpe et al., 1993), its milder allelic variant occipital horn syndrome (OHS) (Kaler et al., 1994) and a form of X-linked hereditary distal motor neuropathy (dHMNX) (Kennerson et al., 2010). The clinical manifestation of these syndromes differs substantially. While MD may result in lethal neurodegeneration in infancy if left untreated and is not associated with lower motor neuron dysfunction, dHMNX is an adult-onset, non-fatal form of motor neuron disease (MND) that predominantly affects the motor neurons in the peripheral nervous system (PNS). These striking phenotypic differences are the result of the distinctive impact that the mutations in the *ATP7A* gene have on the function of the Cu transporter (Kaler, 2011). Intracellular Cu homeostasis is orchestrated by a large network of proteins in which the role of ATP7A shifts between delivering Cu into the secretory pathway of the cell for incorporation into cuproenzymes and the cellular excretion of the metal to maintain cellular Cu levels below toxic concentrations (Petris et al., 1996; Monty et al., 2005; Nyasae et al., 2007). ATP7A executes this dual function through unique trafficking properties. In the neuronal context, ATP7A trafficking is not only associated with changes in the intracellular Cu concentration but has also been demonstrated to be associated with the activation of synaptic N-methyl-D-aspartate (NMDA) receptors (Schlief et al., 2005).

MD causative mutations (small deletions or insertions, nonsense mutations, splice junction mutations, large gene deletions and missense mutations) lead to a profound reduction in the levels and/or functional capacity of ATP7A to transport Cu across the plasma membrane. Given the crucial roles of Cu in the development and function of the central nervous system (CNS) (El Meskini et al., 2007; D'Ambrosi and Rossi, 2015), the devastating consequences that these mutations have on the affected neonates, in which the levels of Cu within the brain are dramatically reduced, are expected. Our group identified two missense mutations (p.T994I and p.P1386S) in the *ATP7A* gene in two independent large families in which affected males had been diagnosed with dHMNX (Kennerson et al., 2010). This seminal discovery highlighted the importance of Cu biology in maintaining the integrity of motor neurons in the PNS. However, the precise mechanisms by which dysfunctional ATP7A leads to the specific length-dependent axonal degeneration seen in dHMNX patient motor neurons remains unknown.

A mouse model in which *Atp7a* had been specifically deleted in the motor neurons provided important evidence for the role of Atp7a and Cu in the maintenance and function of motor neurons (Hodgkinson et al., 2015). However, this strategy is not able to demonstrate the subtle cellular pathomechanisms of the dHMNX point mutations that lead to axonal degeneration. With the purpose of overcoming this limitation, our group recently generated an *Atp7a* conditional knock-in mouse expressing *Atp7a^T985I^*, the orthologue of the human *ATP7A*^T994I^ identified in dHMNX patients (Perez-Siles et al., 2016). This model provided functional evidence that the p.T994I mutation affects ATP7A intracellular trafficking and affected animals showed altered Cu levels within the PNS and CNS. Despite these findings, affected animals did not show a global degenerative motor phenotype or signs associated with axonal degeneration, reflecting the challenge of using mice for modelling human diseases in general, and for inherited peripheral neuropathies in particular.

In this study, we have used skin fibroblasts from a patient harbouring the p.T994I *ATP7A* mutation to generate patient-specific induced pluripotent stem cell (iPSC)-derived motor neuron cultures. Our *in vitro* human neuronal model for dHMNX has shown that the p.T994I mutation leads to a significant reduction of ATP7A protein levels in the cell body of the patient-derived motor neurons. Although affected motor neurons failed to upregulate the expression of ATP7A when exposed to extracellular Cu, dHMNX-derived cells do not display enhanced susceptibility to Cu-induced toxicity. Additionally, trafficking of ATP7A along the axons in the presence of Cu is not compromised in dHMNX-derived motor neurons, suggesting that an alternative pathomechanism is likely to be responsible for triggering the length-dependent axonal degeneration in these patients. Our data reveal mitochondrial abnormalities and bioenergetic deficits in the patient motor neurons prior to morphological changes associated with axonal damage, suggesting that these metabolic changes precede axonal degeneration in dHMNX.

## RESULTS

### dHMNX patient-derived iPSCs maintain pluripotency and normal karyotype

**Fig. 1.**
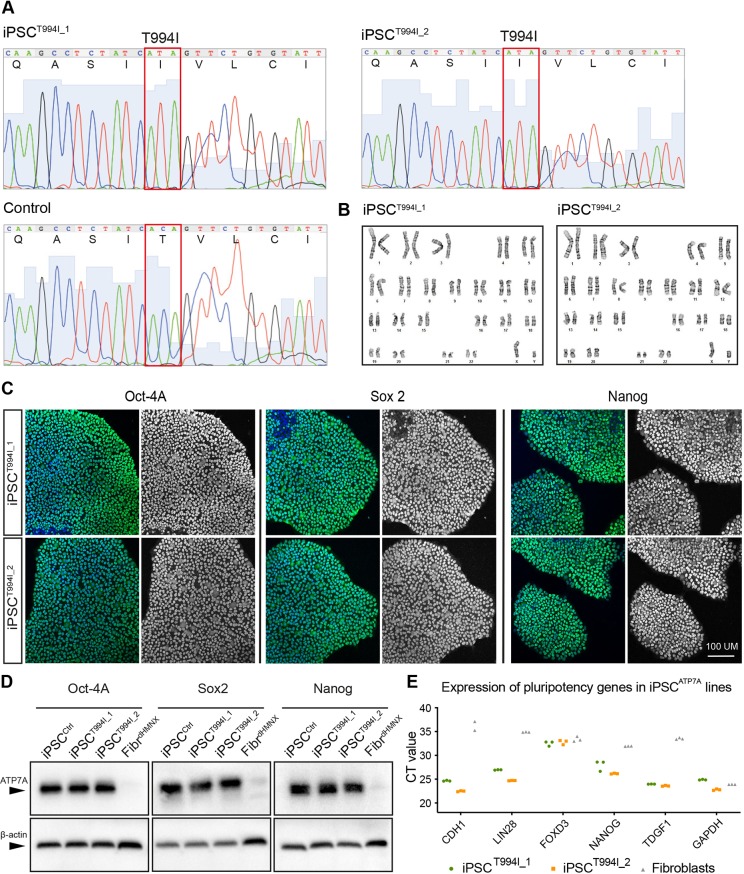
**dHMNX patient-derived iPSCs harbouring the p.T994I ATP7A mutation have a normal karyotype and display cellular features of pluripotency.** (A) Sequencing traces confirm the nucleotide change c.2981C>T in the patient-derived iPSCs. (B) Karyotyping and G-band analysis show that the iPSC clones have a normal 46,XY karyotype. (C) Immunofluorescent staining confirms expression of Oct-4A, Sox2 and Nanog in the two iPSC^T994I^ clones. (D) Western blot demonstrates expression of pluripotency markers in the iPSCs and not in the dHMNX patient fibroblasts. (E) Real-time PCR shows increased expression of *CDH1*, *LIN28*, *NANOG* and *TDGF1* genes in iPSC^T994I_1^ (green) and iPSC^T994I_2^ (orange) lines relative to skin fibroblasts (grey).

Skin fibroblasts from the dHMNX patient harbouring the p.T994I *ATP7A* mutation were reprogrammed using non-integrative episomal plasmids by FUJIFILM Cellular Dynamics following company in-house protocols. The presence of the p.T994I mutation (nucleotide change c.2981C>T) was confirmed by genomic DNA sequencing (Fig. 1A). Karyotyping and G-banding analysis showed the two clones generated from the re-programming (iPSC^T994I_1^ and iPSC^T994I_2^) maintained a normal 46,XY karyotype (Fig. 1B). Pluripotent characteristics of the iPSC lines were confirmed by three experimental approaches. iPSC colonies stained positive for the pluripotency markers Oct-4A, Sox2 and Nanog (Fig. 1C), and protein expression of these transcription factors was absent (Fig. 1D) in the original patient fibroblast lines (Fibr^dHMNX^). Reverse-transcription quantitative real-time PCR (RT-qPCR) revealed that endogenous pluripotency-associated genes (*CDH1*, *LIN28*, *FOXD3*, *NANOG* and *TDGF1*) were expressed at higher levels in the iPSCs when compared to the Fibr^dHMNX^ cells (Fig. 1E).

### Patient-derived iPSC lines display pathogenic features associated with dHMNX patient fibroblasts

**Fig. 2.**
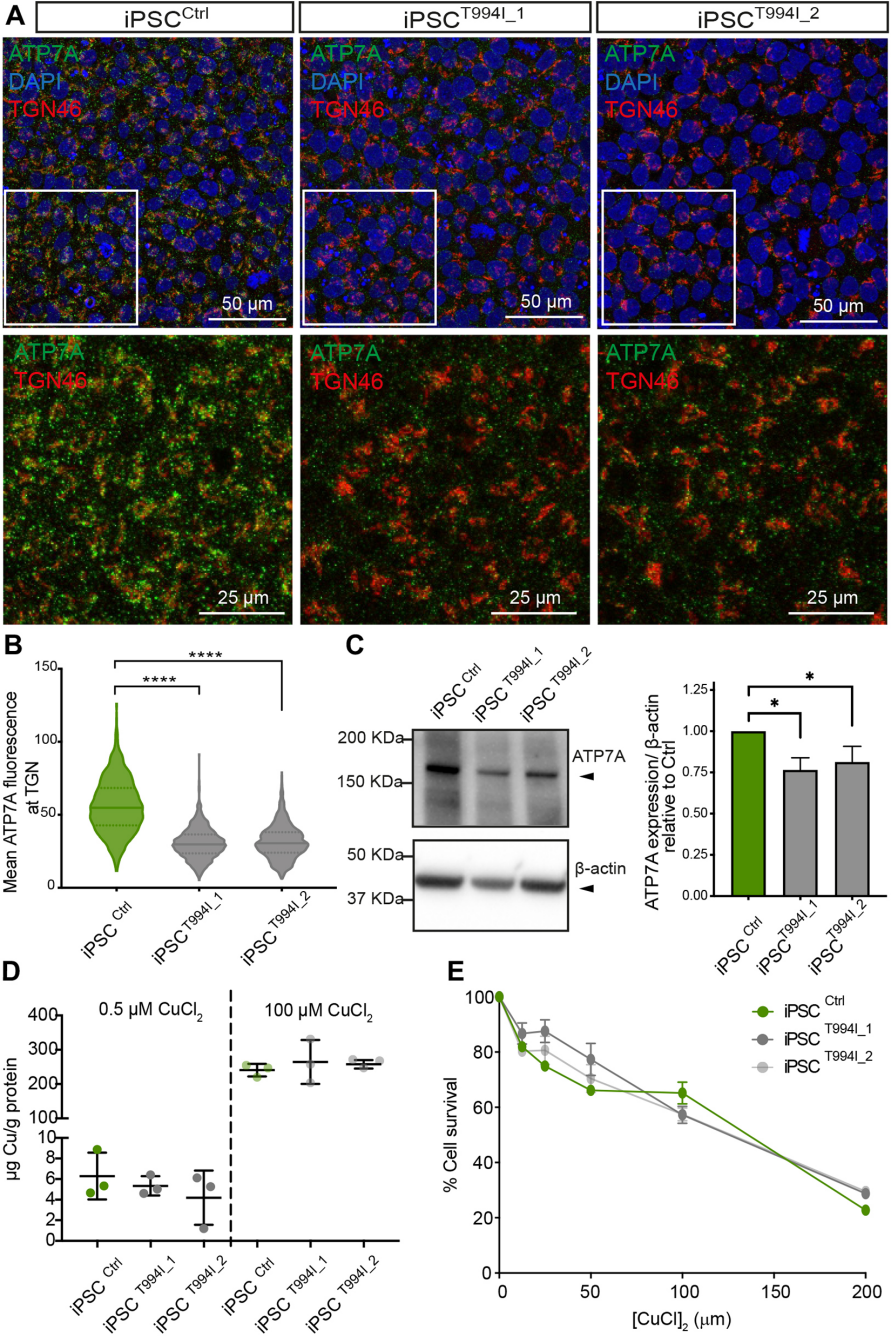
**Assessment of disease phenotypes in dHMNX patient-derived iPSCs.** (A) Staining of iPSC clones shows decreased levels of ATP7A within the trans-Golgi (TGN46) in the patient cells. The boxed areas are enlarged and the DAPI channel removed to highlight increased levels of ATP7A in control iPSCs. (B) Quantification of the ATP7A mean fluorescence within the region of interest (ROI; defined by staining the cells with anti-TGN46 antibody) in patient and control iPSC lines. Violin plot shows full distribution of all data points acquired (*n*>1000 ROIs) and *P*-values were obtained by ANOVA followed by Tukey's post hoc test (****P<0.0001). (C) Levels of ATP7A in protein lysates determined by western blot analysis. Data in bar graphs are represented as mean±s.e.m. and *P*-values were obtained from a two-tailed Student's *t*-test (*P<0.05). (D) Cu content of iPSC pellets was measured in triplicate by ICP-MS following incubation of iPSCs with 0.5 µM and 100 µM CuCl_2_ for 6 h. Data are expressed as the mean±s.e.m. (E) Cu-induced toxicity determined by CCK-8 assay (*n*=4) after culturing iPSCs in a range of CuCl_2_ concentrations, from 0 to 200 μM, for 6 h.

Our previous investigations had shown dHMNX patient fibroblasts have reduced levels of ATP7A localizing at the trans-Golgi network (TGN). Although dHMN is an adult-onset syndrome, this phenotype was also observed in embryonic fibroblasts from a conditional knock-in mouse expressing the dHMNX-causative mutation (Perez-Siles et al., 2016). Staining of iPSCs showed a pattern of intracellular distribution for ATP7A in both the cytoplasm and within the TGN in control iPSCs, with reduced levels of ATP7A in the patient-derived iPSCs (Fig. 2A,B). Assessing the total levels of ATP7A protein by western blot analysis (Fig. 2C) confirmed decreased expression of ATP7A under basal Cu conditions (0.5 µM CuCl_2_) in the iPSC^T994I^ clones when compared with the iPSC^Ctrl^. Despite the reduced protein expression of ATP7A in the iPSC^T994I^ clones, patient-derived cells did not accumulate intracellular Cu under basal or Cu loading conditions (Fig. 2D). Accordingly, no differences were found between the iPSC^T994I^ and iPSC^Ctrl^ lines when Cu-induced toxicity was assessed (Fig. 2E).

### Differentiation of dHMNX-derived iPSCs into spinal cord motor neurons

**Fig. 3.**
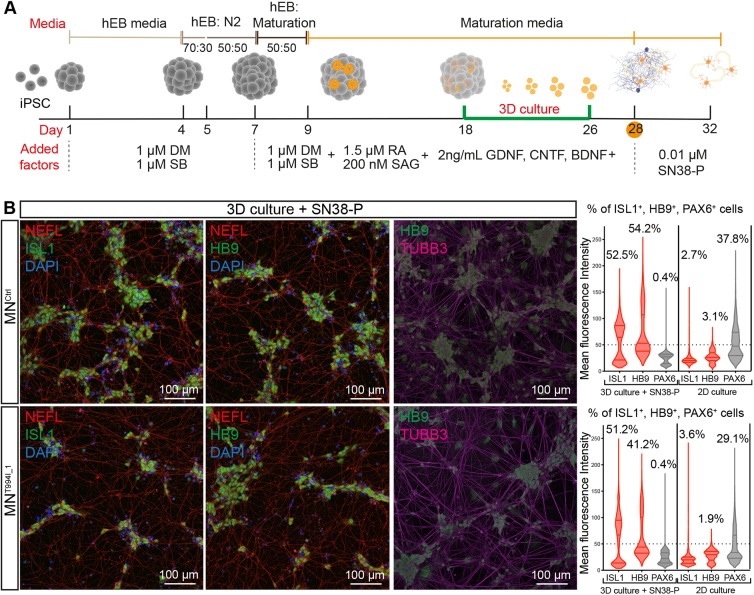
**Differentiation of dHMNX patient-derived and control iPSCs into motor neurons.** (A) Timeline for spinal cord motor neuron differentiation from iPSCs following a dual SMAD inhibition protocol. Diagram shows media used (see Materials and Methods for full description) and factors added throughout the process. Cells were grown in suspension (3D) between days 18 and 26. DM, dorsomorphin; SB, SB431542; RA, retinoic acid; SAG, Smoothened agonist; GDNF, glial-cell-line-derived neurotrophic factor; CNTF, human ciliary neurotrophic factor; BDNF, brain-derived neurotrophic factor; SN38-P, phosphorylated 7-ethyl-10-hydroxycamptothecin; hEB, human embryoid body medium. (B) Differentiated motor neurons show a robust expression of ISL1, HB9, βIII-tubulin (TUBB3) and NEFL markers at day 32. Maturation of motor neurons in suspension followed by incubation with SN38-P enriches for ISL1/HB9-expressing cells and reduces the proportion of PAX6^+^ cells. Mean fluorescence intensity of the indicated markers within all nuclei (DAPI) was determined and cells with values above 50 units (threshold empirically determined for each marker) were considered positive for ISL1, HB9 or PAX6, respectively. Violin plot shows full distribution of all data points acquired (*n*>3000 nuclei).

dHMNX patients develop muscle weakness and wasting due to axonal degeneration of motor neurons (Kennerson et al., 2013). To develop a neuronal model for dHMNX, iPSC^T994I^ and iPSC^Ctrl^ lines were differentiated into adherent spinal cord motor neurons using protocols that were initially reported by Chambers et al. (2009) and subsequently modified by Saporta et al. (2015) to generate neuronal models of axonal Charcot-Marie-Tooth neuropathy (CMT), a group of hereditary neuropathies involving both the motor and sensory neurons of the PNS. For this protocol, neuralization of the iPSCs is achieved by dual SMAD signalling inhibition (dorsomorphin and SB431542) and neuronal patterning triggered with retinoic acid (RA) and activation of the Sonic hedgehog (SHH) pathway with Smoothened agonist (SAG). Later addition of glial-cell-line-derived neurotrophic factor (GDNF), ciliary neurotrophic factor (CNTF) and brain-derived neurotrophic factor (BDNF) collectively contribute to mature motor neuron development (Fig. 3A).

Our protocol has been slightly modified and maturation of differentiating motor neurons takes place in a three-dimensional (3D) culture system (days 18-26). This additional step has been adapted from Shimojo and colleagues (2015) and has recently been applied to patient-derived iPSC lines for modelling CMT (Sainio et al., 2018). In these conditions, differentiating motor neurons aggregate on suspended spheres (spheroids). On day 26 of the *in vitro* differentiation protocol (26 DIV), spheroids are dissociated into single cells and cultured in the presence of 0.01 μM SN38-P from 28 DIV onwards for the removal of proliferative stem cells (Mao et al., 2018). Within 1 week of adherent differentiation, immunofluorescence analysis revealed the efficient induction of HB9 and ISL-1, markers for somatic motor neurons (Fig. 3B and Fig. S1A). The presence of these markers was significantly upregulated in the suspension 3D culture following the SN38-P treatment when compared with the monolayer differentiation process (∼50% versus ∼3%, respectively), where PAX6^+^ cells are enriched (∼0.4% versus ∼35%; Fig. S1B). Differentiated motor neurons also expressed neuronal cytoskeletal proteins NEFL and βIII-tubulin (Fig. 3B). This analysis revealed no differences in the expression of the analyzed markers and showed similar neurite structures in patient (MN^T994I^) and control (MN^Ctrl^) motor neurons, indicating that the differentiation potential of the dHMNX-derived motor neurons and their ability to establish neuronal networks is not affected by the *ATP7A* mutation. Further maturation of MN^Ctrl^ and MN^T994I^ (45 DIV) showed no effect on axonal integrity or additional signs of degeneration (Fig. S1C).

### Expression of ATP7A is downregulated in dHMNX-derived motor neurons

**Fig. 4.**
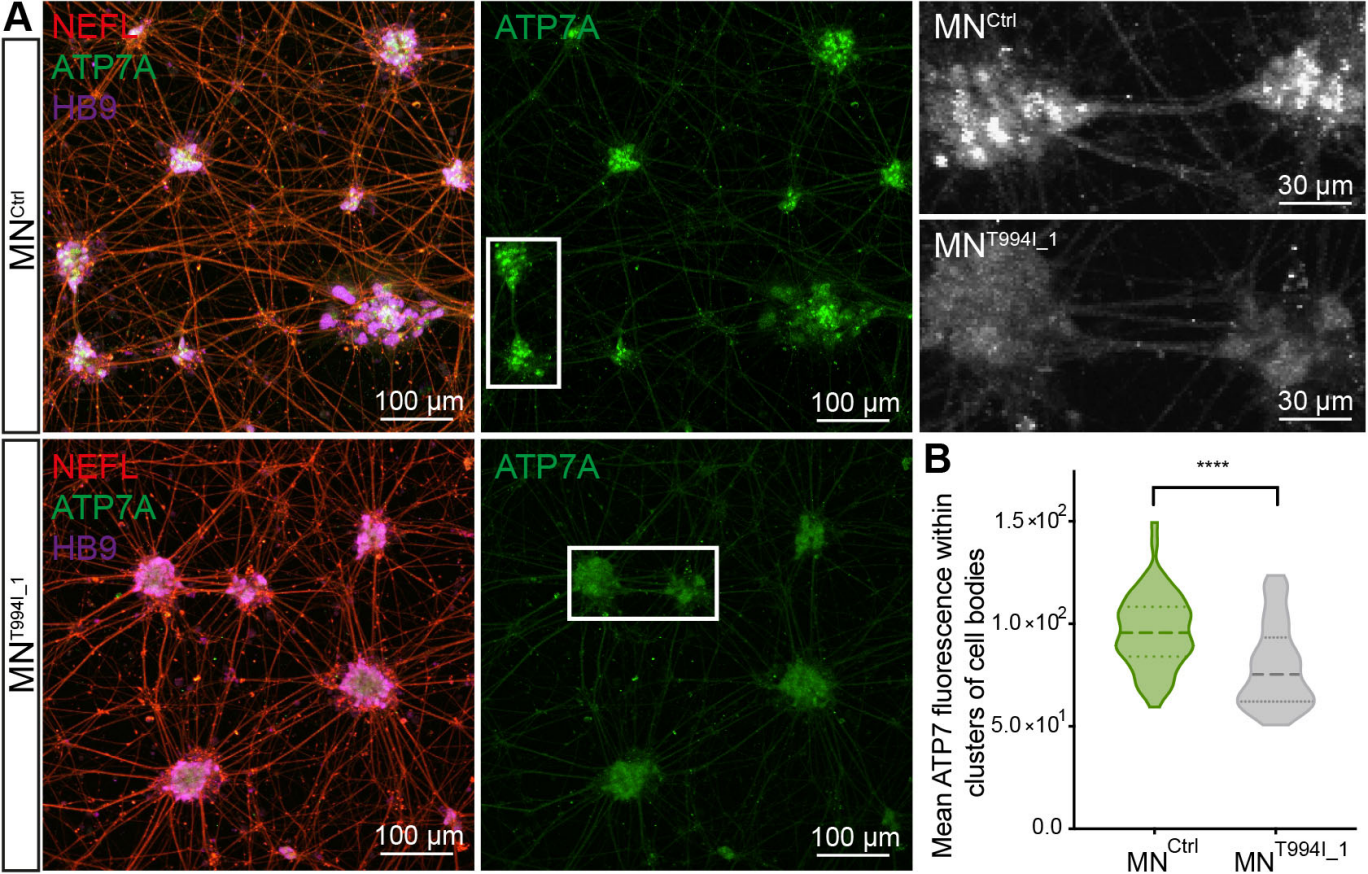
**ATP7A protein level is reduced at the soma of dHMNX-derived motor neurons.** (A) Staining of motor neurons at 32 DIV shows ATP7A localizing predominantly in the soma of control cells (MN^Ctrl^) in basal conditions and a substantial reduction in ATP7A levels in the patient-derived motor neurons (MN^T944I^). Boxed area is enlarged with the ATP7A staining shown in greyscale. (B) Quantification of the ATP7A mean fluorescence within the clusters of cell bodies (defined by staining of the nuclei with HB9) confirmed a statistically significant reduction (two-tailed Student's *t*-test, ****P<0.0001) in ATP7A levels in MN^T994I^ clones. Violin plot shows full distribution of all data points acquired (*n*>100 ROIs).

We next investigated whether ATP7A protein was differentially expressed between the patient and control motor neurons. After 7 days of adherent differentiation, cells organized into clusters that contain motor neuron cell bodies, with extending axons connecting these structures (Figs 3B and 4A). Staining with an anti-ATP7A antibody at 32 DIV and observation at low magnification (20×) showed a decrease in the levels of ATP7A within the cell bodies of patient motor neurons (Fig. 4A and Fig. S2A). Using the HB9 staining, we defined the areas of the cultures occupied by clusters of cell bodies and quantified the levels of ATP7A under basal Cu conditions. Using this approach, we found a significant decrease in the levels of ATP7A of dHMNX-derived motor neurons within these clusters, while no difference was observed in the axonal compartment (Fig. 4B). Motor neurons differentiated from an additional iPSC line [derived from an X-linked CMT type 6 (CMTX6) patient] that expresses wild-type *ATP7A* showed expression of ATP7A equivalent to MN^Ctrl^. This result indicates that the reduction in the levels of ATP7A protein seen in the dHMNX motor neurons is a disease-specific phenotype (Fig. S2B).

### Cu-induced ATP7A trafficking and Cu homeostasis is not affected in dHMNX-derived motor neurons

**Fig. 5.**
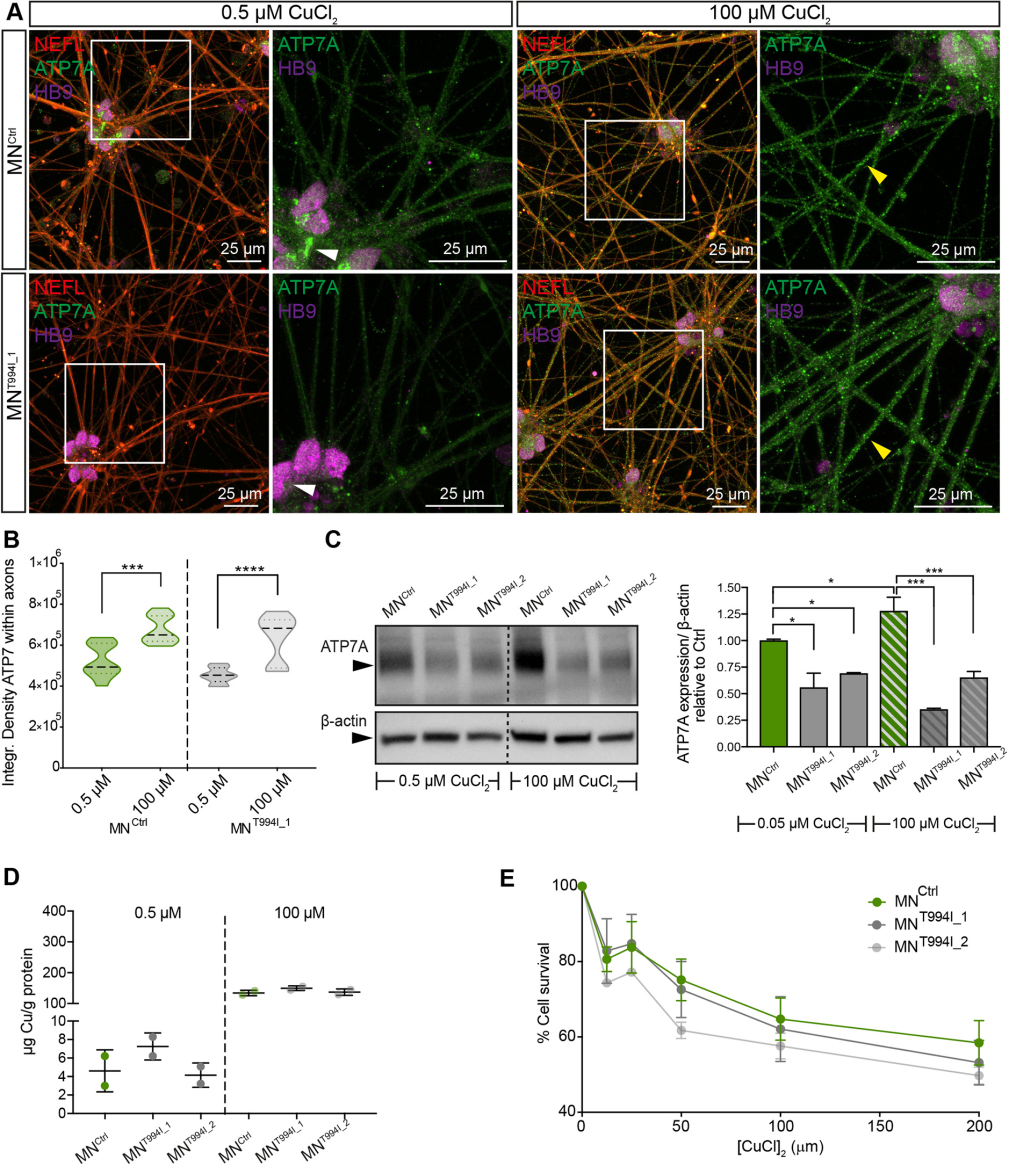
**Response of dHMNX patient-derived motor neurons to increased CuCl_2_.** (A) ATP7A traffics from the soma (white arrowhead) to axons (yellow arrowhead) in control (MN^Ctrl^) and patient-derived (MN^T944I^) motor neurons after 3 h incubation with 100 µM CuCl_2_. (B) Quantification of the ATP7A mean fluorescence at 32 DIV within axons (defined by staining the cells with NEFL) confirms Cu-induced trafficking of ATP7A in both MN^Ctrl^ and MN^T944I^ motor neurons. Violin plot shows full distribution of all data points acquired (*n*>50 images) and *P-*values were obtained by ANOVA followed by Tukey's post hoc test (***P<0.001, ****P<0.0001). (C) ATP7A protein levels are reduced in the MN^T994I^ clones. Incubation of motor neurons with 100 μM CuCl_2_ increases the levels of ATP7A protein exclusively in the control cells. Vertical dashed line separates samples from the same western blot assembled for this figure. Data in bar graphs are represented as mean±s.e.m. and *P-*values were obtained from a two-tailed Student's *t-*test (*P<0.05, ***P<0.001). (D) Cu content of motor neuron pellets was measured by ICP-MS. Data are expressed as the mean±s.e.m. (E) Cu-induced toxicity determined by CCK-8 assay (*n*=4) after culturing motor neurons in a range of CuCl_2_ concentrations, from 0 to 200 μM, for 6 h.

ATP7A trafficking properties in response to intracellular Cu levels have been extensively investigated. However, Cu-dependent mobilization of wild-type and mutant forms of ATP7A in motor neurons have not been studied under physiological conditions due to the lack of the appropriate research models. Differentiated control and dHMNX-derived motor neurons were cultured under basal (0.5 µM CuCl_2_) and high (100 µM CuCl_2_) Cu levels, and the distribution of ATP7A assessed by confocal microscopy at 32 DIV. Observation at high magnification (63×) showed ATP7A perinuclear localization in the soma of control motor neurons under basal conditions. In contrast, this perinuclear localization was not observed in the patient cells under the same experimental settings (Fig. 5A and Fig. S2B). Previous studies showed that overexpressing the ATP7A p.T994I mutant protein in the spinal cord/neuroblastoma hybrid cell line (NSC-34) led to mutant ATP7A re-localizing from the TGN compartments to the cell body's plasma membrane under basal Cu conditions and to the axonal plasma membrane after NSC-34 differentiation (Yi et al., 2012). Under our more physiological conditions, however, ATP7A^T994I^ is not observed at the plasma membrane of axons nor cell bodies in the presence of low Cu. When we cultured iPSC-derived motor neurons in the presence of high levels of Cu, ATP7A extensively localized to axonal compartments in both control and patient-derived cells (Fig. 5A and Fig. S2B). In our experimental approach, individual axons are bundled together so we are unable to determine the precise axonal localization of ATP7A (axonal plasma membrane versus axoplasm). Quantification of ATP7A levels in the axonal compartment confirmed the visual observation and showed no difference between control and dHMNX-derived motor neurons under basal or high Cu levels (Fig. 5B), suggesting that Cu-induced trafficking is not affected by the p.T994I mutation.

Western blot analysis of protein lysates from differentiated motor neurons showed a significant decrease in ATP7A levels in the patient cells. Intracellular Cu accumulation increases steady-state ATP7A protein levels through stabilization of the protein by Cu (Xie and Collins, 2013). Using patient fibroblasts and primary cells from our *Atp7a^T985I^* knock-in mouse model, we had shown that the dHMNX mutation impairs this post-translational mechanism of ATP7A regulation (Perez-Siles et al., 2016). Our experiments using iPSC-derived motor neurons confirm that exposure to Cu increases ATP7A protein levels in control cells and motor neurons cultured from our additional CMTX6-derived iPSC line (20%), which serves as an additional control. This effect is absent in the dHMNX-derived motor neurons (Fig. 5C and Fig. S2C). Despite the reduction in the ATP7A protein levels in the patient motor neurons, the levels of ATP7A available in the mutant cells are sufficient to maintain Cu homeostasis under basal and Cu loading conditions (Fig. 5D) with no increased Cu-induced toxicity observed in the dHMNX lines at DIV 32 (Fig. 5E). Further maturation/aging of the iPSC-derived motor neurons (DIV 45) did not affect this result, suggesting an alternative mechanism(s) to defective Cu transport and mobilization of ATP7A in response to Cu as a dHMNX pathomechanism (Fig. S1D).

### Mitochondrial features and bioenergetics are affected in dHMNX-derived motor neurons

**Fig. 6.**
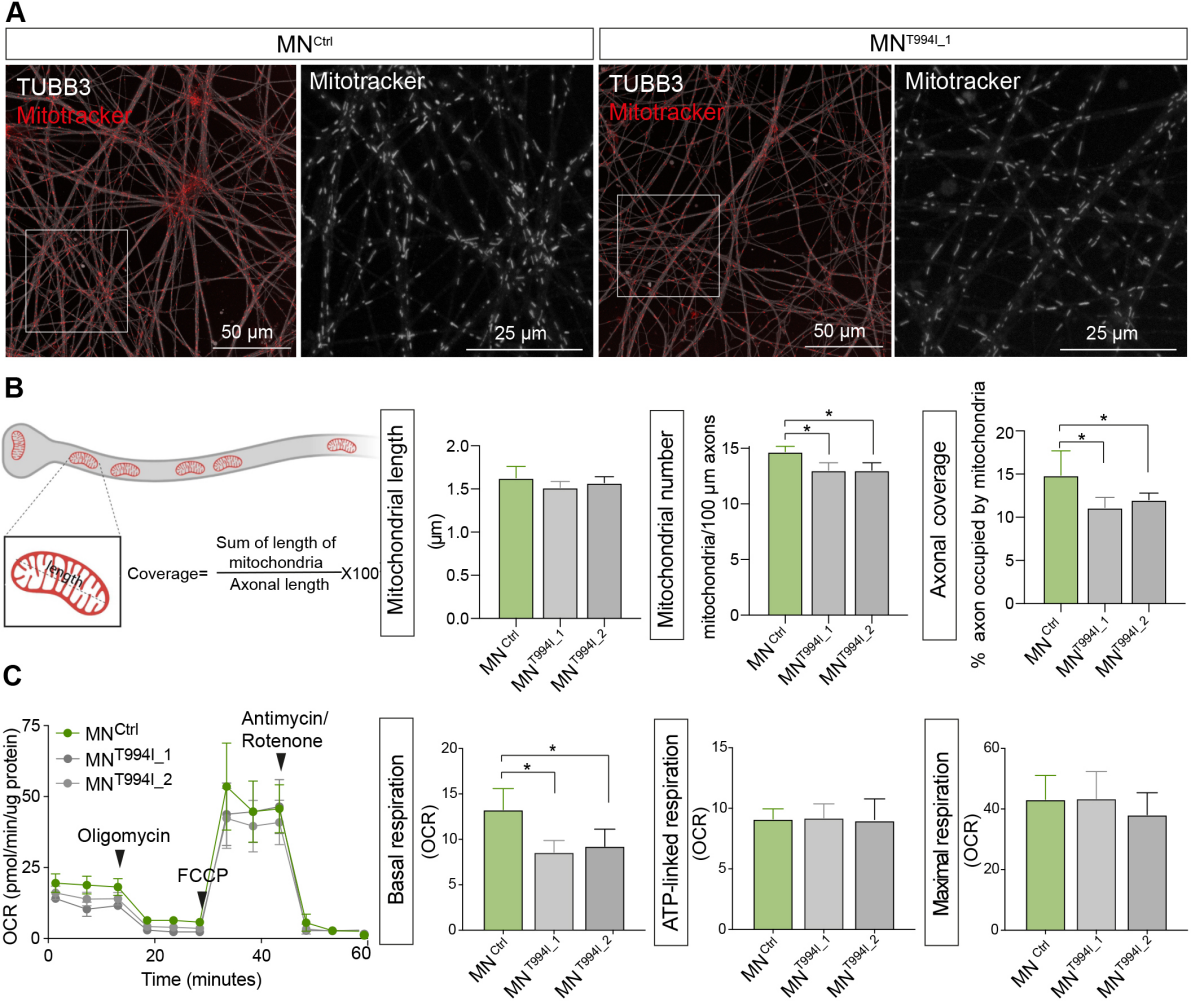
**Mitochondrial features and bioenergetics in dHMNX patient-derived motor neurons.** (A) Motor neurons stained with MitoTracker and TUBB3. Boxed area is shown enlarged in the right panel. (B) Morphological features of mitochondria were assessed by length and number of mitochondria within axons. Axonal coverage was calculated by dividing the total length of all stained mitochondria by the total length of the axons. (C) Overview of the oxygen consumption rate (OCR) throughout the mitochondrial respiration test using the Seahorse XF24 Cell Mito Stress Kit. Arrowheads indicate the time when mitochondrial inhibitors were added to the media to assess respiratory parameters. OCR prior to the addition of oligomycin determines basal respiration and shows energetic demand of the cell under baseline conditions. ATP-coupled oxygen consumption was determined by inhibiting ATP synthase using oligomycin. Maximal respiration was assessed following mitochondria uncoupling by FCCP. The error bars indicate the mean±s.e.m. of five replicates of one representative experiment. *P*-values were obtained by ANOVA followed by Tukey's post hoc test (*P<0.05).

Delivery of Cu into the mitochondria is fundamental for cellular respiration. To determine whether mitochondrial features were affected by the *ATP7A* mutation, iPSC-derived motor neurons were stained with MitoTracker (Fig. 6A), and morphological features of individual mitochondria determined in MN^Ctrl^ and MN^T944I^ clones (Fig. 6B). Although the mean length of mitochondria was the same between control and patient-derived cells, the number of mitochondria as well as the axonal coverage (proportion of axons occupied by mitochondria) was found to be significantly reduced in the dHMNX-derived motor neurons. To assess the functional impact of these differences, the bioenergetic profiles of control and dHMNX-derived motor neurons at DIV 45 were obtained by measuring the oxygen consumption rate (OCR) using an extracellular flux analyzer (Fig. 6C). Data from these experiments showed a decrease in baseline respiration in the MN^T994I^ clones when compared to MN^Ctrl^. Addition of specific respiratory complex inhibitors revealed no significant differences in the ATP-linked respiration or maximal respiration between these lines. The absence of signs of axonal fragmentation at DIV 45 (Fig. S1C) suggests that changes in mitochondrial features and metabolic deficits associated with the p.T994I *ATP7A* mutation precede axonal damage in dHMNX.

## DISCUSSION

Along with the neurodegeneration characteristic of Menkes disease, Cu dysregulation has been recognized as a pathological feature of neurodegenerative diseases in which axonal degeneration occurs, such as Alzheimer's disease (AD), Parkinson's disease (PD) and amyotrophic lateral sclerosis (ALS) (reviewed by Giampietro et al., 2018). Mutations in the gene encoding the Cu transporter ATP7A provided the first evidence for the importance of Cu homeostasis in maintaining the integrity of the PNS (Kennerson et al., 2010). Despite the growing evidence supporting the role of Cu in the pathomechanisms underlying axonal degeneration in dHMN (Merner et al., 2011), to date no therapies for this group of diseases are available to patients, in part due to the lack of relevant disease models (Juneja et al., 2019). Rodent models have yielded important insights into some molecular mechanisms of disease development and/or facilitated the development of promising treatment strategies for neurodegenerative diseases in general, and inherited peripheral neuropathies in particular. However, these efforts have rarely translated into clinical application for reasons that may include basic differences in the anatomy, physiology and genetic background of human and mouse. Our recently developed conditional knock-in mouse model for dHMNX (*Atp7a^T985I^*) proved the dysregulation of Cu homeostasis within the PNS and CNS of affected mice (Perez-Siles et al., 2016). However, these animals did not develop axonal degeneration, providing an additional example that supports the need for developing complementary model systems to help elucidate the cellular processes that lead to human disease.

In the present study, we successfully established a dHMNX iPSC model from a patient harbouring the p.T994I mutation in the *ATP7A* gene. Using previously described protocols (Saporta et al., 2015) with modifications introduced by our laboratory and a final purification using SN38-P (Mao et al., 2018), we have obtained patient-derived motor neurons that recapitulate disease-associated phenotypes found in the dHMNX fibroblasts. The patient-derived motor neurons described here represent the first available human neuronal model to assess the link between Cu-dependent pathways and degeneration of motor neurons in patients.

**Fig. 7.**
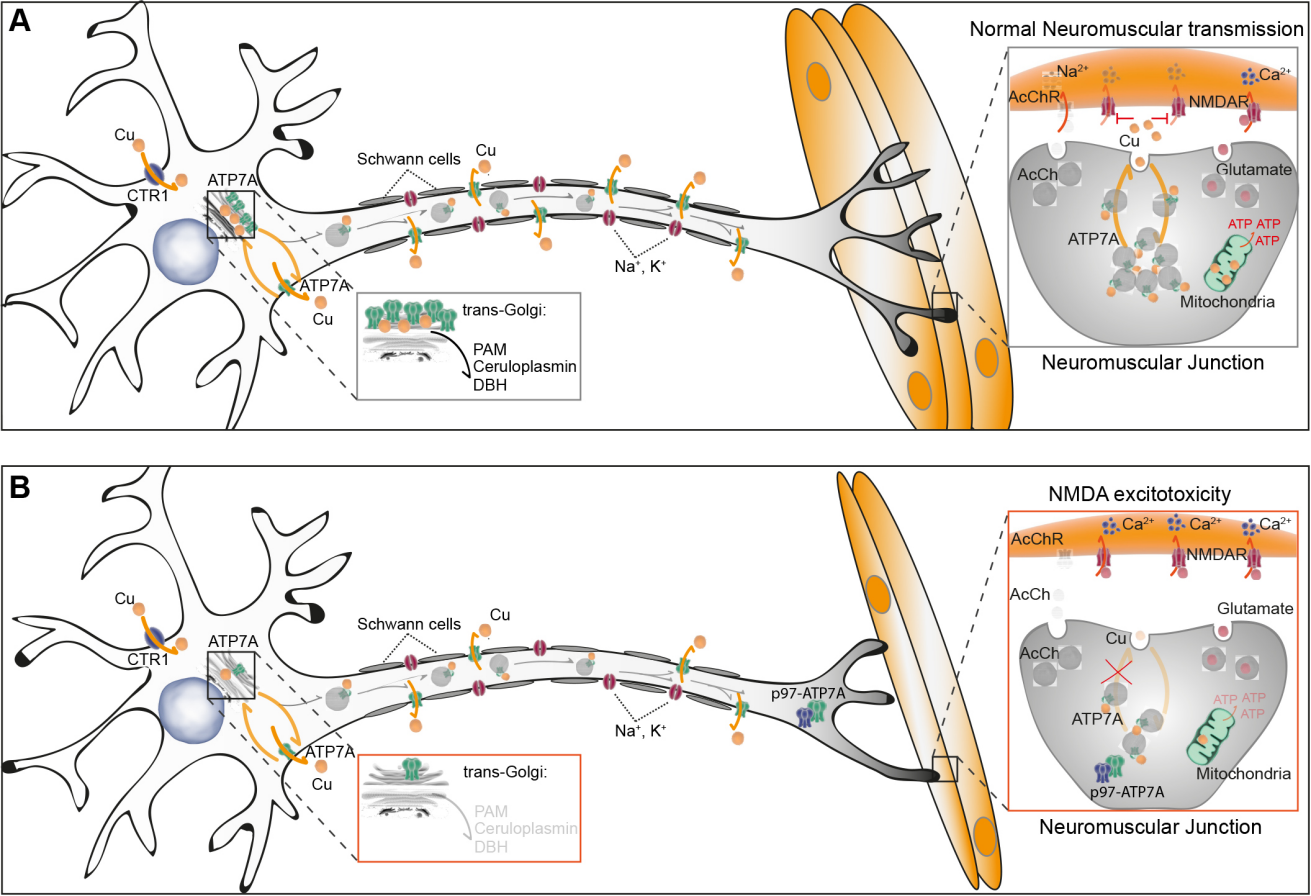
**Working hypotheses of X-linked distal hereditary motor neuropathy (dHMNX) mechanism.** (A) In control cells, ATP7A resides in the soma of motor neurons, where it localizes at the trans-Golgi network (TGN) until copper (Cu) triggers release from this compartment and anterograde ATP7A trafficking. At the synapse, glutamate-activated NMDA receptors are regulated by Cu ions released at the neuromuscular junction (NMJ) by ATP7A. (B) In mutant motor neurons, supply of Cu to cuproenzymes by ATP7A at the trans-Golgi, regulation of synaptic activity by Cu and/or mitochondrial metabolism may be affected by the dHMNX p.T994I mutation. ATP7A, Cu-transporting ATPase; CTR1, Cu transporter 1; PAM, peptidyl α-amidating monooxygenase; DBH, dopamine β-hydroxylase; p97, ATPase valosin-containing protein; AcCh, acetyl choline; AcChR, acetyl choline receptor; NMDAR, N-methyl-D-aspartate receptor.

Our experiments show that ATP7A protein levels in the dHMNX motor neurons do not increase in response to Cu loading as do levels of the wild-type protein (Fig. 5C). This phenotype is consistent with our previous findings obtained using skin fibroblasts from this patient and also in the primary cells and neuronal tissues from our dHMNX mouse model (Perez-Siles et al., 2016). In the highly polarized structure of spinal cord motor neurons, our data show ATP7A with a perinuclear localization at the soma of control cells under basal Cu levels. In these conditions, the levels of ATP7A detected in the soma of patient motor neurons are significantly reduced. As summarized in the model depicted in Fig. 7, ATP7A at the trans-Golgi provides Cu to Cu-dependent enzymes. In the neuronal context, peptidyl α-amidating monooxygenase (PAM), dopamine β-hydroxylase (DBH) and ceruloplasmin are cuproenzymes whose impaired activity is associated with defective synaptic function and/or neurodegeneration.

PAM is essential to the biosynthesis of approximately half of all neuropeptides (Eipper and Mains, 1988). *PAM*^+/−^ mice develop neurological deficits associated with enhanced excitability and deficient synaptic plasticity. This phenotype was recapitulated in Cu-restricted wild-type mice and rescued in Cu-supplemented *PAM*^+/−^ mice (Gaier et al., 2014). DBH catalyzes the oxidative hydroxylation of dopamine to norepinephrine and is expressed both in the brainstem and in peripheral nerve terminals. Several groups have reported the association of specific polymorphisms in the *DBH* gene and reduced DBH activity with susceptibility to PD (Ghosh et al., 2019) and AD (Mustapic et al., 2013), respectively. The importance of iron homeostasis in the PNS, in which the ferroxidase enzyme ceruloplasmin plays a central role, has been extensively reviewed (Levi and Taveggia, 2014). Metallation of PAM, DHB and ceruloplasmin relies on sufficient ATP7A at the TGN (Fig. 6A). Hence, reduced availability of the Cu transporter in this location might be a potential pathomechanism underlying axonal degeneration in dHMNX patients (Fig. 6B). The dHMNX-derived motor neurons presented here are an ideal model to assess this hypothesis.

Two ground-breaking papers revealed a neuroprotective role for Cu that could be the key mechanism explaining the pathogenesis underlying dHMNX. Activation of the NMDA receptor in mouse primary hippocampal neurons caused Cu-independent trafficking of Atp7a to neuronal processes, which was associated with a rapid release of Cu from the neurons (Schlief et al., 2005). This study also showed that the released Cu was neuroprotective against NMDA-mediated excitotoxic cell death and was directly linked to a Cu-induced reduction in calcium influx (Fig. 6A). In a follow-up study, primary hippocampal neurons cultured from the *Atp7a*-deficient *Mo^Br^* mouse were more sensitive to glutamate-mediated NMDA-receptor-dependent excitotoxicity (Schlief et al., 2006). NMDA receptors have been found at the postsynaptic end plate of neuromuscular junctions (NMJs) (Mays et al., 2009), suggesting a regulatory function of glutamate and NMDA receptors at the vertebrate NMJ. Based on these findings, it is feasible that the ATP7A^T994I^ mutation may affect synaptic release of Cu induced by NMDA activation and hence cause progressive neuronal death through a mechanism of NMDA-mediated excitotoxicity (Fig. 6B). Further experiments are required to assess whether trafficking of ATP7A and Cu in response to NMDA activation is impaired in the patient-derived cells. Although our data suggest that Cu-induced ATP7A trafficking is not affected in dHMNX-derived motor neurons (Fig. 5A,B), our experimental approach utilizes a 3 h incubation of the cells with CuCl_2_. In these conditions, the whole pool of ATP7A available for the cells may relocalize to axonal compartments and minimize a subtle trafficking defect associated with the p.T994I mutation. Additional experiments using shorter time points of CuCl_2_ loading will confirm whether Cu-induced trafficking is affected in the dHMNX-derived motor neurons.

Although global Cu homeostasis appears not to be affected in patient motor neurons (Fig. 5D), our current model lacks the metabolic and functional support of the myelin sheath. The major challenge facing iPSC studies for inherited peripheral neuropathies is the availability of protocols that can differentiate iPSCs into fully functional myelinating Schwann cells. Although the neurophysiology studies of patients with the p.T994I mutation show an axonal rather than a demyelinating neuropathy (Kennerson et al., 2010), the role of myelination in Cu exchange and homeostasis cannot be excluded in the patient motor neurons. Additionally, subcellular distribution and availability of Cu within the polarized structure of motor neurons may be disrupted by the mutation. Recent publications have reported the efficient separation of neuronal somatodendritic and axonal compartments in iPSC-derived motor neurons (Maciel et al., 2018). Using this approach, we will be able to determine whether subcellular Cu distribution is affected in our dHMNX neuronal model.

The increasing number of inherited peripheral neuropathy genes associated with mitochondrial biology (Pareyson et al., 2015) suggests that the maintenance of bioenergetic homeostasis is crucial for structural and functional integrity of distal axons. The discovery of mutations in the cuproenzymes *SCO2* (Rebelo et al., 2018) (mitochondrial Cu-binding protein) and *COX6A1* (Tamiya et al., 2014) (cytochrome c oxidase subunit 6A1) as genetic causes for specific subtypes of axonal CMT supports the hypothesis that reduced availability of Cu to the respiratory chain within the mitochondria is a pathological mechanism in dHMNX. The mitochondrial abnormalities and bioenergetic deficits seen in the patient motor neurons highlights the opportunity to interrogate a possible functional connection between the p.T994I *ATP7A* mutation, mitochondrial dysfunction and axonal degeneration using our dHMNX neuronal model.

There are over 370 *ATP7A* mutations causing Menkes disease (Tümer, 2013). The majority of these mutations (75%) result in a nonfunctional truncated protein or nonsense-mediated decay mechanism. Lethal infantile neurodegeneration seen in Menkes disease patients is therefore attributable to ATP7A loss of function. A possible gain-of-function mechanism caused by the p.T994I *ATP7A* mutation may specifically affect peripheral nerves in dHMNX patients. In support of this idea, previous research had shown that the p.T994I mutation causes an aberrant interaction of the Cu transporter with valosin-containing protein (VCP/p97) not observed in the wild-type ATP7A (Yi et al., 2012). Mutations in *VCP* have been reported to account for a spectrum of phenotypes, including an axonal form of CMT (CMT2Y) (Gonzalez et al., 2014) and ALS (ALS14) (Johnson et al., 2010). The identified CMT2Y causative *VCP* missense mutation (p.E97K) led to impaired autophagic function. By using the dHMNX-derived motor neurons we will be able to investigate whether the increased interaction of mutant ATP7A with VCP/p97 leads to axonal degeneration in dHMNX through a similar toxic gain-of-function mechanism.

This study has presented a patient-derived motor neuron model for dHMNX that recapitulates pathological features previously identified in patient fibroblasts and a rodent model for this disease. Further investigations utilizing the MN^T994I^ model will allow elucidation of the precise mechanisms by which this mutation produces axonal degeneration in dHMNX patients. Furthermore, dHMNX-derived motor neurons can be added to the list of *in vitro* cellular models that serve as a tool to study common mechanisms of pathogenesis for inherited peripheral neuropathies and the importance that ATP7A and Cu-dependent pathways have in other neurodegenerative diseases.

## MATERIALS AND METHODS

### Research guidelines and regulations

All research and cell culture procedures were conducted following written consent, according to protocols approved by the Sydney Local Health District Human Ethics Review Committee, Concord Repatriation General Hospital, Sydney, Australia (reference number HREC/11/CRGH/105). Informed consent for study participation was obtained from all patients and controls. All research was performed in accordance with relevant guidelines and regulations.

### Patient iPSC generation

Reprogramming was performed by FUJIFILM Cellular Dynamics as previously described (Yu et al., 2011). Briefly, fibroblasts obtained from a dHMNX patient harbouring the p.T994I mutation were transfected using oriP/EBNA-1-based vectors (Yu et al., 2009) and then placed on Matrigel-coated plates in reprogramming medium (Yu et al., 2011) for 1 week followed by Essential 8™ (E8) medium for an additional 2 weeks. The iPSC colonies were individually picked and propagated with E8 on Matrigel-coated plates. Absence of genetic abnormalities following the re-programming process was confirmed by karyotype analysis of the iPSC clones (WiCell). The pluripotency of the iPSC lines was confirmed by assessing expression of endogenous pluripotent stem cell genes and the identity of the iPSCs was matched to the starting fibroblast line (FUJIFILM Cellular Dynamics).

### Motor neuron differentiation

The motor neuron differentiation protocol is based on dual SMAD inhibition and was performed as previously described (Saporta et al., 2015) with modifications introduced by our laboratory to increase efficacy of the procedure. Briefly, colonies were expanded as adherent cultures on Matrigel-coated culture plates to 90-95% confluence. Cells were then switched to knockout serum replacement medium containing 1 μM dorsomorphin dihydrochloride (STEMCELL Technologies) and 10 μM SB431542 (STEMCELL Technologies) and, after 3 days, gradually changed to N2 medium. On day 7, cultures were changed to a motor neuron maturation medium consisting of DMEM/F12 supplemented with B27, N2, 0.8 mM ascorbic acid, 1.5 μM retinoic acid, 200 nM SAG (EMD Chemicals), 2 ng/ml BDNF, 2 ng/ml CTNF and 2 ng/ml GDNF (Life Technologies), and matured for 10 days. Cultures were then dissociated using 1 U/ml dispase (STEMCELL Technologies) and terminal differentiation achieved by a 3D culture system using ultra-low attachment in six-well plates (Corning-Costar) for an additional 12 days with replacement of the maturation media every second day. Maturation of motor neurons in these conditions facilitated aggregation of cells in homogeneous spheres (spheroids) that were subsequently dissociated into single cells using Accutase and subsequently seeded on Matrigel-coated plates for final maturation in the presence of 0.01 μM SN38-P to decontaminate the culture of proliferative stem cells (Mao et al., 2018).

### Immunofluorescence

Cells were washed once using PBS, fixed with 4% paraformaldehyde (PFA) for 12 min at room temperature (RT), permeabilized in phosphate-buffered saline (PBS) containing 0.3% (v/v) Triton X-100 and blocked in 5% (w/v) bovine serum albumin (BSA) for 60 min. Cells were incubated with primary antibodies overnight in 4°C. The antibodies used for immunofluorescence staining are as follows: anti-SOX2 (Cell Signaling Technology, #3579, 1:400), anti-NANOG (Cell Signaling Technology, #4903, 1:400), anti-OCT4 (Cell Signaling Technology, #2840, 1:400), anti-TUBB3 (Sigma-Aldrich, T2220, 1:500), anti-NEFL (Abcam, 8186, 1:1000), anti-ISL1 [Developmental Studies Hybridoma Bank (DSHB), 40.2D6, 1:150], anti-PAX6 (DSHB, 81.5C10, 1:200), anti-HB9 (Millipore, ABN174, 1:500), anti-Golgin 97 (Santa Cruz Biotechnology, CDF4, 1:500) and anti-ATP7A antibody raised against the C-terminal 18 amino acids (NH2-DKHSLLVGDFREDDDTAL-COOH) of human ATP7A (Antibody Solutions, 1:500).

The following Alexa Fluor (AF) secondary antibodies were purchased from Invitrogen: AF-488 goat anti-rabbit IgG (A-11008), AF-488 goat anti-mouse IgG (A-11001), AF-555 goat anti-mouse IgG (A-21422), AF-555 goat anti-rabbit IgG (A-21428) and AF-647 goat anti-chicken IgY (A-21449). All secondary antibodies were used at 1:500 dilution and incubated for 2 h at RT. Nuclei were stained with 300 nM 4,6-diamidino-2-phenylindole (DAPI; Molecular Probes). When required, cells were incubated with 100 nM MitoTracker Deep Red FM (Invitrogen) for 45 min to visualize mitochondrial structures. Samples were mounted using Prolong Gold antifade reagent (Invitrogen).

### RT-qPCR

RNA was extracted from confluent iPSC colonies using the RNeasy mini kit (Qiagen). RNA (1 μg) was reverse transcribed using the High-Capacity cDNA Reverse Transcription Kit (Applied Biosystems). Quantitative RT-PCR was performed using TaqMan Gene Expression Assays using the following probes: *CDH1* (Hs01023895_m1), *LIN28* (Hs00702808_s1), *FOXD3* (Hs00255287_s1), *NANOG* (Hs02387400_g1), *TDGF1* (Hs02339499_g1) and *GAPDH* (Hs02786624_g1). To qualitatively assess the differential expression of the pluripotency genes in the iPSC lines versus the dHMNX patient fibroblasts, raw Ct values are presented.

### Western blotting

Cell lysates were obtained from confluent iPSC lines using RIPA buffer (50 mM Tris-HCl pH 8.0, 150 mM NaCl, 0.1% w/v SDS, 1% v/v Triton X-100, 1% w/v sodium deoxycholate, 1× cOmplete Mini EDTA-free protease inhibitor). After quantitating the amount of protein (Pierce BCA Protein Assay Kit, Thermo Fisher Scientific), 15 μg of cell lysates were subjected to SDS-polyacrylamide gel electrophoresis and transferred to polyvinylidene difluoride (PVDF) membranes. Membranes were probed with anti-SOX2 (Cell Signaling Technology, #3579, 1:400), anti-NANOG (Cell Signaling Technology, #4903, 1:400), anti-OCT4 (Cell Signaling Technology, #2840, 1:400) and anti-ATP7A (Antibody Solutions, 1:250) antibodies. β-actin (Cell Signaling Technology) was used as loading control. Anti-rabbit (Sigma-Aldrich) and anti-mouse (Abcam) horseradish peroxidase (HRP)-conjugated secondary antibodies were used and signal detected by adding a chemiluminescent substrate (Merck).

### Cytotoxicity assays

Cell viability of iPSCs and differentiated motor neurons was determined with the CCK8 assay kit (Sigma-Aldrich). Following a 6 h exposure to increasing concentrations of CuCl_2_, cells were washed with PBS and incubated with CCK8 diluted 1:10 (v/v) in E8 (iPSCs) or DMEM/F12 (motor neurons) for 90 min at 37°C. The absorbance at 450 nm was measured with an EnSpire Multimode Plate Reader (Perkin Elmer). Data were obtained from three independent experiments. Each data point is represented as the mean±s.e.m. for four experimental replicates per concentration of CuCl_2_.

### Intracellular metal analysis

Total intracellular metal content was measured in the iPSC and motor neuron pellets using inductively coupled plasma mass spectrometry (ICP-MS; Agilent 7700, Varian). Using six-well plates, iPSC colonies were cultured to confluency and 2×10^5^ iPSC-derived motor neurons (after dissociation with Accutase) were grown for 5 days (in the presence of SN38-P 0.01 μM). Cells were then treated with 0.5 μM CuCl_2_ or 100 μM CuCl_2_ in culture medium for 6 h at 37°C. After the treatments, cells were harvested, an aliquot saved for protein determination (Pierce BCA Protein Assay Kit, Thermo Fisher Scientific) and pellets dissolved in 70% nitric acid as previously described (Hodgkinson et al., 2015) for determining total intracellular metal concentration. The mean value with standard errors (±s.e.m.) determined in triplicate for each test condition was used for comparison.

### Bioenergetic assessment of iPSC-derived motor neurons

Mitochondrial OCRs were measured using a XF24 Seahorse Biosciences Extracellular Flux Analyzer. Following differentiation of the iPSC-derived motor neurons, 75,000 cells per well were plated onto a Matrigel-coated Seahorse 24-well plate, and maturation continued with the above-indicated medium until the day of the experiment (DIV 45). At 45 min prior to initiating the assay, maturation medium was replaced with 500 µl of XF unbuffered medium supplemented with 10 mM glucose, 1 mM pyruvate and 2 mM L-glutamine, and the cells were incubated at 37°C to allow medium temperature and pH to reach equilibrium. Seahorse analyzer injection ports contained (A) 1 µM oligomycin A; (B) an ATP synthase inhibitor to inhibit OXPHOS and test respiration coupling to ATP synthesis; (C) 1 µM FCCP, a protonophore uncoupling agent to increase respiration rate and enable the quantification of the maximal respiration under maximal mitochondrial uncoupling; and (D) 1 µM rotenone/antimycin, inhibitors of mitochondrial respiratory complex I and complex III, respectively, to cause complete inhibition of mitochondrial respiration and hence determine maximal mitochondrial OCR. Normalization of the recorded data was carried out after quantifying total protein content per well and data represented as OCR per µg protein.

### Statistical analysis and representation of data

For the statistical analysis, three independent experiments under the same conditions were performed and a Student's *t*-test or one-way ANOVA followed by Tukey's post hoc test used to assess the significance of the results. The data are expressed as mean±s.e.m. *P*-values <0.05 were considered statistically significant. The following statistical thresholds have been applied throughout the study: *P<0.05; **P<0.01; ***P<0.001, ****P<0.0001. All data were plotted using GraphPad Prism (v8). To show the full distribution of all points acquired in immunofluorescence experiments, data are shown as violin plots where indicated.

This article is part of a special collection ‘A Guide to Using Neuromuscular Disease Models for Basic and Preclinical Studies’, which was launched in a dedicated issue guest edited by Annemieke Aartsma-Rus, Maaike van Putten and James Dowling. See related articles in this collection at http://dmm.biologists.org/collection/neuromuscular.

## Supplementary Material

Supplementary information
