# Supplementary material for: Modelling the pathogenesis of X-linked distal hereditary motor neuropathy using patient-derived iPSCs
Source: Dis Model Mech. 2020 Jan 13;13(2):dmm041541. doi: 10.1242/dmm.041541 (PMC6994953; doi:10.1242/dmm.041541)
Supplement: Supplementary information [file dmm-13-041541-s1.pdf]

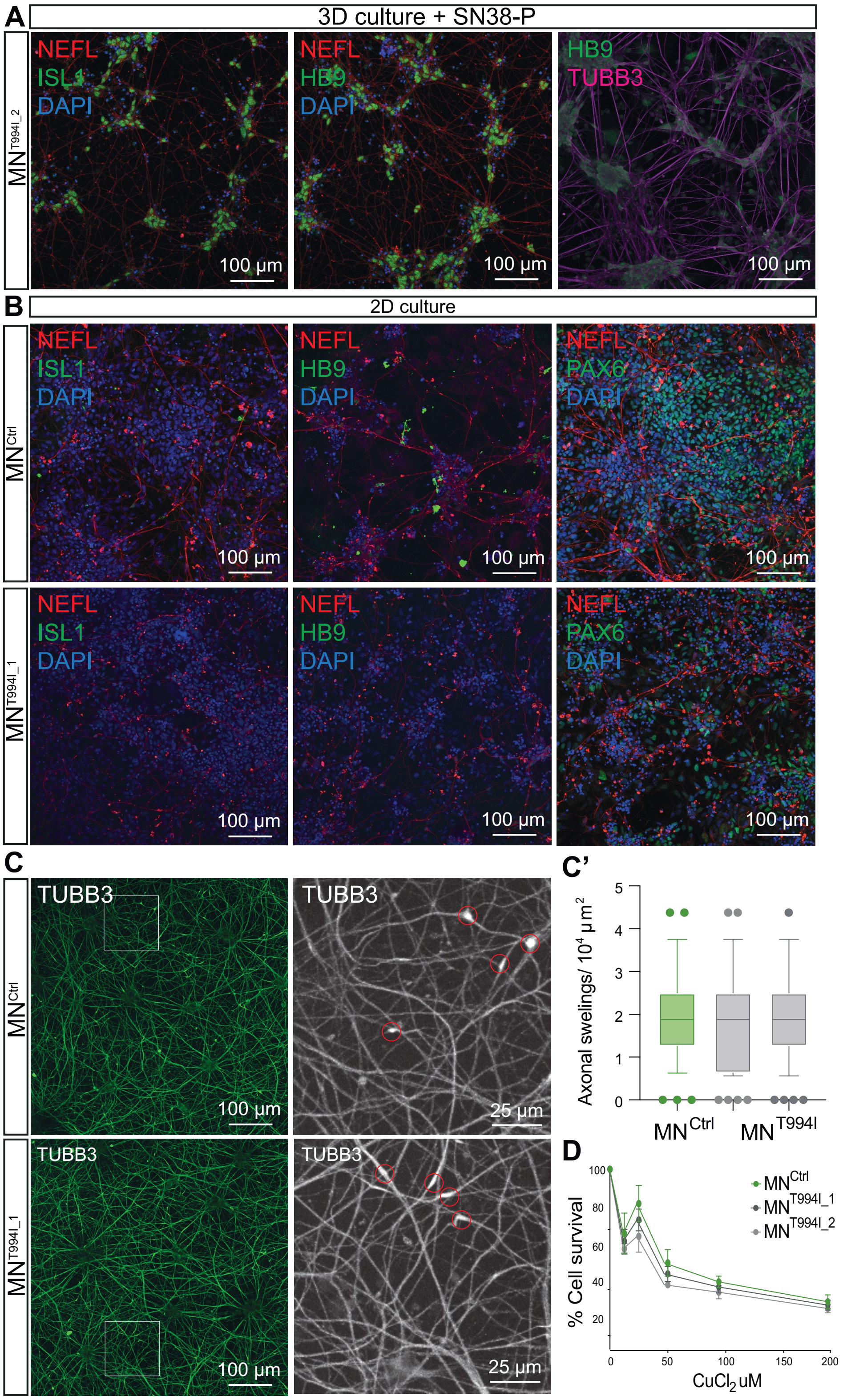

Fig S1. A. MN^{T994I_2} differentiated motor neurons from additional clone show a robust expression of ISL1, HB9, TUBB3 and NEFL markers at day 32. **B.** Maturation of motor neurons in 2D and no purification using SN38-P results in enrichment of the culture in MN^{Ctrl} and MN^{T994I} cells expressing PAX6^{+} cells. **C.** MN^{Ctrl} and MN^{T994I} clones at 45 DIV show no signs of axonal fragmentation. Boxed area is enlarged with the TUBB3

Disease Models & Mechanisms • Supplementary information

staining shown in grayscale to highlight swelling of axons. **C'.** Appearance of axonal swelling was assessed by applying a threshold to the TUBB3 staining so that enlarged areas of the neurites were highlighted. These structures were automatically counted by restricting the parameters of the "Analyze particles" tool in Image J to particles bigger than 3 μm^2 and circularity between 0.6 - 1.0. **D.** Cu-induced toxicity at DIV 45 determined by CCK-8 assay (n=4) after culturing motor neurons to a range of $CuCl_2$ concentrations from 0 μM to 200 μM for 6 h.

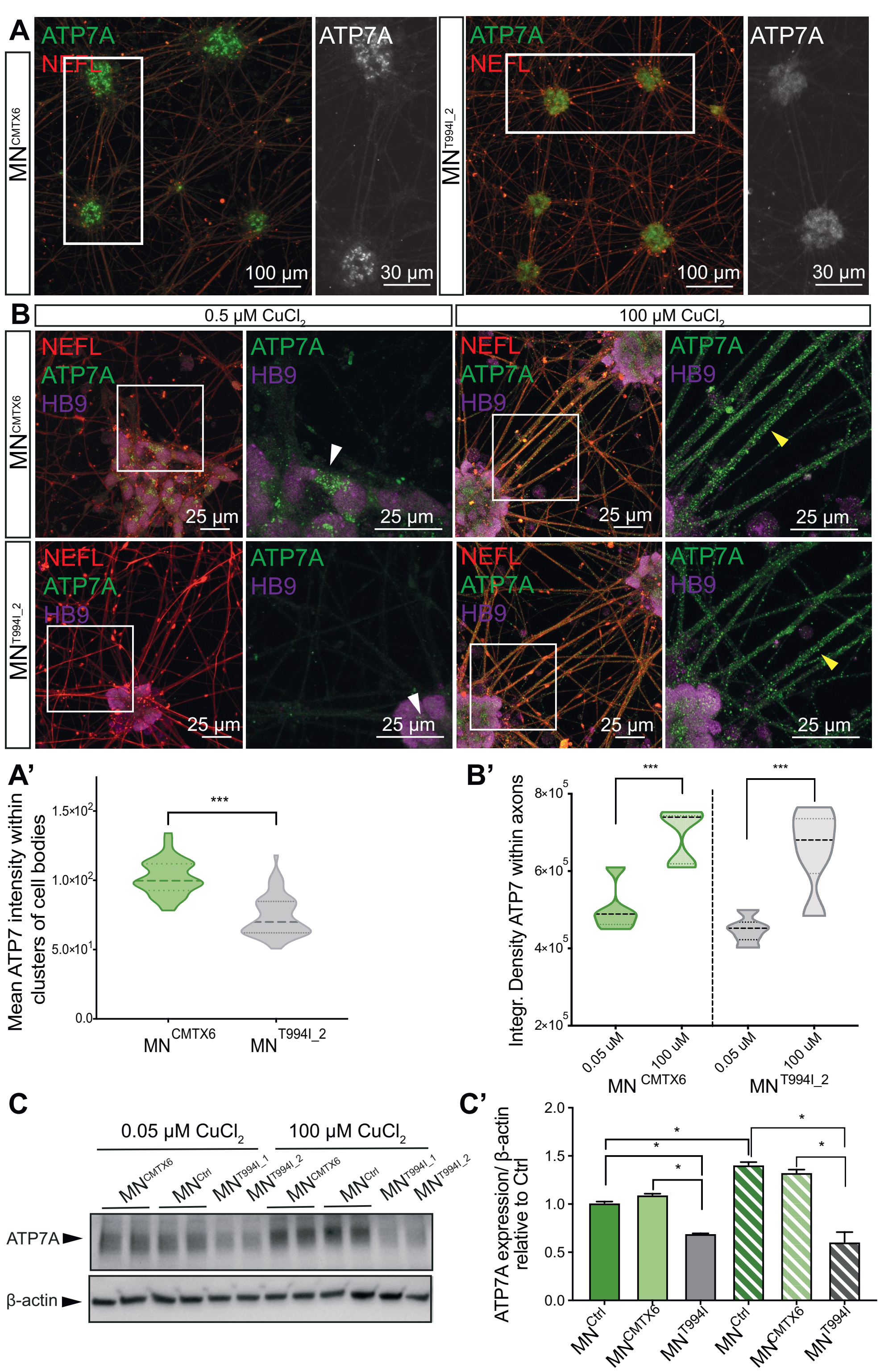

Fig S2. A. Staining of motor neurons shows ATP7A localising predominantly in the soma of motor neurons cultured from a CMTX6-derived iPSC line (MN^{CMTX6}) in the absence of added $CuCl_2$ and a strong reduction of the transporter in the additional clone from dHMNX patient-derived motor neurons (MN^{T944I_2}). **A'.** Quantification of the ATP7A mean fluorescence within the clusters of cell bodies confirms a statistically significant reduction of ATP7A levels in MN^{T994I_2}. Violin plot shows full distribution of

all data points acquired (n > 100 ROIs). **B.** ATP7A traffics from the soma (white arrow) to axons (yellow arrow) in CMTX6 and dHMNX patient derived motor neurons after 3 hours incubation with 100 μM $CuCl_2$. **B'.** Quantification of the ATP7A mean fluorescence at 32 DIV within axons (defined by staining the cells with NEFL) confirms Cu-induced trafficking of ATP7A in both MN^{CMTX6} and MN^{T944I_2} motor neurons. Violin plot shows full distribution of all data points acquired (n > 50 images) and p values were obtained by ANOVA followed by Tukey's post hoc test. **C.** ATP7A protein levels are reduced in the MN^{T994I} clones when compared to motor neurons expressing wild type ATP7A (MN^{Ctrl} and MN^{CMTX6}). Incubation with 100 μM $CuCl_2$ increases the levels of ATP7A protein exclusively in the MN^{Ctrl} and MN^{CMTX6} cells. **C'.** Data in bar graphs are represented as mean ± SEM and p values were obtained from a two-tailed Student's t test.
